# Supplementary material for: Plant Secondary Metabolites in the Battle of Drugs and Drug-Resistant Bacteria: New Heroes or Worse Clones of Antibiotics?
Source: Antibiotics (Basel). 2020 Apr 10;9(4):170. doi: 10.3390/antibiotics9040170 (PMC7235868; doi:10.3390/antibiotics9040170)
Supplement: Supplementary file 1 [file antibiotics-09-00170-s001.zip › antibiotics-757085-supplementary.docx]

Table S1. Anti-biofilm activity of SMoPs.

| **Substance** | **Group** | **Pathogen** | **Concentration** | **Mechanism** | **Ref.** |
| --- | --- | --- | --- | --- | --- |
| Eugenol | phenylpropanoids | *P. aeruginosa* | 150-300 μg/ml | inhibition and reducing pre-formed biofilms;  down-regulation the expression of biofilm-associated genes; inhibition of QS | [162, 125] |
|  |  | *L. monocytogenes* | 2,5mM | interruption in cell-cell communication | [163-164] |
|  |  | *K. pneumoniae* | 63,5 μg/ml |  | [132, 124] |
|  |  | *MRSA* |  |  | [137] |
| Cinnamaldehyde |  | *L. monocytogenes* | 0,75 mM | disturbance of cellular integrity | [163] |
|  |  | *S. epidermidis* | 125 μg/ml |  | [165-166] |
| Gallic acid | benzoic acid derivatives | *P.aeruginosa* | 200 μg/ml | inhibition of QS | [167] |
|  |  | *P. aeruginosa* | 25 μg/ml |  | [129] |
| Ellagic acid |  | *E. coli, S. aureus* | 15- 40 μg/ml | inhibition of QS | [168] |
| 1,2,3,4,6-penta-O-galloyl-β-D-glucopyranose | tannins | *S. aureus* | 4 μM | inhibition of the initial attachment to solid surface; inhibition of the synthesis of polysaccharide intercellular adhesin | [139] |
| Punicalagin |  | *S. typhimurium* | 15.6 μg/ml | down-regulation of QS and motility-related genes | [169] |
| Resveratrol | stilbenoids | *P.aeruginosa, E.coli* | 50 μg/ml | inhibition of QS | [128] |
|  |  | *S. aureus* | 100 μg/ml |  | [138] |
| Quercetin | flavonoids | *E. coli, S. aureus* | 1 μg/ml | reduction of the expression of QS genes | [124] |
| Curcumin | diarylheptanoids | *P.aeruginosa* | 1 μg/ml |  | [170, 131] |
|  |  | *S. epidermidis,  E. coli,  Proteus mirabilis,  Serratia marcescens,  K. pneumoniae,  Enterococcus faecalis,  S. mutans* | 25-625 μg/ml |  | [134, 165, 171-173] |
| Thymol | terpenoids | *Listeria monocytogenes* | 5 mM | down-regulation of QS genes | [163] |
|  |  | *Listeria monocytogenes* | 750 μg/ml |  | [174] |
|  |  | *P. aeruginosa* |  |  | [130] |
|  |  | *S. typhimurium and S. enteritidis* | 312 μg/ml | inhibition and reducing pre-formed biofilms; disturbance of cellular integrity | [175] |
| Carvacrol |  | *L. monocytogenes* | 0.65-10 mM | down-regulation of QS genes | [163] |
|  |  | *P. aeruginosa* |  |  | [130] |
|  |  | *P. aeruginosa* |  |  | [176] |
|  |  | *S. typhimurium and S. enteritidis* | 156 μg/ml | inhibition and reducing pre-formed biofilms; disturbance of cellular integrity | [175] |
|  |  | *S. enterica* | 0.33 mg/ml |  | [177] |
| Betulinic acid |  | *P. aeruginosa* | 125 μg/ml | inhibition of QS | [127] |
| Ursolic acid |  | *P. aeruginosa, E. coli* | 10 μg/ml |  | [178-179] |
| linalool |  | *S. typhimurium* | 0.166 mg/ml | anti-adhesion activity | [180] |
| Allicin | sulfur-containing  phytochemicals | *S. epidermidis* | 128 μg/ml | inhibition of QS | [181-182] |
|  |  | *P. aeruginosa* | 4 mg/ml |  | [126] |
| Aesculetin | coumarin derivatives | *S. aureus* | 128 μg/ml |  | [183] |
|  |  | *E. coli* | 50 μg/ml |  | [124] |
| Umbelliferone |  | *E. coli* | 50 μg/ml |  | [124, 184] |
| Chrysophanol, emodin and shikoninall | quinones | *P. aeruginosa* | 20 μM |  | [185] |
| Berberine | alkaloids | *K. pneumoniae* | 63.5 μg/ml | inhibition of QS | [134] |
|  |  | *S. epidermidis* | 30-45 μg/ml |  | [140] |
|  |  | *S. epidermidis* | 125 μg/ml |  | [136] |
| Chelerythrine and sanguinarine |  | *S. aureus* | 15-25 μM |  | [186] |
| Chelerythrine |  | *S. aureus* | 4 μg/ml |  | [135] |
| Chelerythrine and sanguinarine |  | *S. epidermidis* | 5- 9 μM |  | [186] |
| Indole |  | *K. pneumoniae* | 15.6 μg/ml |  | [134] |
|  |  | *K. pneumoniae* | > 255 μM |  | [133] |
| p-Coumaric acid | cinnamic acid derivative | *S. typhimurium* | 0.104 mg/ml | anti-adhesion activity | [180] |

Table S2. Synergistic interactions of some SMoPs with antibiotics.

| **Pathogen** | **Drug** | **MIC, μg/ml** | **+ SMoP** | **MICs reduction** | **Ref.** |
| --- | --- | --- | --- | --- | --- |
| *E. coli; S. aureus; P. aeruginosa* | Chloramphenicol | 128; 8; 1024 | + essential oils from *Dittrichia graveolens (L.) Greuter* | 10-fold | [187] |
| *S. aureus; P. aeruginosa* | Imipenem | 4 | + *Ocimum asilicum* essential oil | 32-fold | [188] |
| *E. feacium* | Tetracycline | 256 | + carvacrol | 4-fold | [189] |
|  | Tetracycline | 256 | + thymol | 4-fold | [189] |
| *E. coli (MDR)* | Tetracycline | 200 | + chanoclavine | 8-to-16-fold | [190] |
|  | Tetracycline | 200 | + lysergol | 4-fold | [190] |
|  | Tetracycline | 200 | + piperine | 4-to-8-fold | [190] |
| *S. aureus* | Norfloxacin | 156.3 | + gallic acid | 3-fold | [191] |
|  | Gentamicin | 49.21 | + gallic acid | 20-fold | [191] |
|  | Rifampicin | > 256 | + kaempferol or quercetin | 4-fold | [192] |
|  | Ciprofloxacin | 6.25 | + kaempferol rhamnoside | 4-fold | [193] |
|  | Tetracycline and norfloxacin | 64 | + 6-geranyl coumarin | 2-fold | [194] |
|  | Tetracycline | 128 | + eugenol | 4-fold | [189] |
|  | Tetracycline | 128 | + carvacrol | 2-fold | [189] |
|  | Tetracycline | 4 | + carvacrol or thymol | 2-to-8-fold | [189] |
|  | Ciprofloxacin | 0.10 | + reserpine | 4-fold | [193] |
| *MRSA* | Norfloxacin | >100 | + bergamottin epoxide | 20-fold | [195] |
|  | Gentamicin | 32 | + piperine | 4-fold | [62] |
| *MDR clinical isolates of S. aureus* | Ciprofloxacin and tetracycline | 10-80 | + galbanic acid | 4-to-16- fold | [196] |
| *S. epidermidis* | Amoxicillin | 16 | + quercetin | 4-fold reduction | [197] |

Reference

1. Zhou, L.; Zheng, H.; Tang, Y.; Yu, W.; Gong, Q. Eugenol inhibits quorum sensing at sub-inhibitory concentrations. *Biotechnol. Lett*. **2013**, *35*, 631–663, doi:10.1007/s10529-012-1126-x.
2. Upadhyay, A.; Upadhyaya, I.; Kollanoor-Johny, A.; Venkitanarayanan, K. Antibiofilm effect of plant derived antimicrobials on Listeria monocytogenes. *Food Microbiol*. **2013**, *36*, 79–89, doi:10.1016/j.fm.2013.04.010.
3. Mohammed, S.A. Removal of Listeria monocytogenes biofilm with some local plant extracts of Kurdistan region, Iraq. *ARO* **2019**, *7*, 1–4, doi:10.14500/aro.10385.
4. Sharma, G.; Raturi, K.; Dang, S.; Gupta, S.; Gabrani, R. Combinatorial antimicrobial effect of curcuminwith selected phytochemicals on Staphylococcus epidermidis. *J. Asian Nat. Prod. Res.* **2014**, *16*, 535–541, doi:10.1080/10286020.2014.911289.
5. Albano, M.; Crulhas, B.P.; Alves, F.C.B.; Pereira, A.F.M.; Andrade, B.F.M.T.; Barbosa, L.N.; Furlanetto, A.; Lyra, L.P.S.; Rall, V.L.M.; Júnior, A.F. Antibacterial and anti-biofilm activities of cinnamaldehyde against S. epidermidis. *Microb. Pathog.* **2019**, *126*, 231–238, doi:10.1016/j.micpath.2018.11.009.
6. Bali, E.B.; Türkmen, K.E.; Erdönmez, D.; Sağlam, N. Comparative study of inhibitory potential of dietary phytochemicals against quorum sensing activity of and biofilm formation by Chromobacterium violaceum 12472, and swimming and swarming behaviour of Pseudomonas aeruginosa PAO1. *Food Technol. Biotechnol*. **2019**, *57*, 212–221, doi:10.17113/ftb.57.02.19.5823.
7. Bakkiyaraj, D.; Nandhini, J.R.; Malathy, B.; Pandian, S.K. The anti-biofilm potential of pomegranate (Punica granatum L.) extract against human bacterial and fungal pathogens. *Biofouling* **2013**, *29*, 929–937, doi:10.1080/08927014.2013.820825.
8. Kiran, M.D.; Adikesavan, N.V.; Cirioni, O.; Giacometti, A.; Silvestri, C.; Scalise, G.; Ghiselli, R.; Saba, V.; Orlando, F.; Shoham, M.; et al. Discovery of a quorum-sensing inhibitor of drug-resistant staphylococcal infections by structure-based virtual screening. *Mol. Pharmacol*. **2008**, *73*, 1578–1586, doi:10.1124/mol.107.044164.
9. Rudrappa, T.; Bais, H.P. Curcumin, a known phenolic from curcuma longa, attenuates the virulence of Pseudomonas aeruginosa PAO1 in whole plant and animal pathogenicity models. *J. Agric. Food Chem.* **2008**, *56*, 1955–1962, doi:10.1021/jf072591j.
10. Hu, P.; Huang, P.; Chen, M.W. Curcumin reduces Streptococcus mutans biofilm formation by inhibiting sortase A activity. *Arch. Oral Biol.* **2013**, *58*, 1343–1348, doi:10.1016/j.archoralbio.2013.05.004.
11. Neelakantan, P.; Subbarao, C.; Sharma, S.; Subbarao, C.V.; Garcia-Godoy, F.; Gutmann, J.L. Effectiveness of curcumin against Enterococcus faecalis biofilm. *Acta Odontol. Scand*. **2013**, *71*, 1453–1457, doi:10.3109/00016357.2013.769627.
12. Packiavathy, I.A.S.V.; Priya, S.; Pandian, S.K.; Ravi, A.V. Inhibition of biofilm development of uropathogens by curcumin—an anti-quorum sensing agent from Curcuma longa. *Food Chem*. **2014**, *148*, 453–460, doi:10.1016/j.foodchem.2012.08.002.
13. Centorbi, H.J.; Aliendro, O.E.; Mattana, C.M. Effect of thymol and environmental factors on growth and biofilm formation by Listeria monocytogenes. *Bol. Lat. Caribe Plant. Med. Aromat* **2019**, *18*, 411–424, doi:10.35588/blacpma.19.18.4.26.
14. Amaral, V.C.S.; Santos, P.R.; da Silva, A.F.; dos Santos, A.R.; Machinski, M.Jr.; Mikcha, J.M.G. Effect of carvacrol and thymol on Salmonella spp. biofilms on polypropylene. *Int. J. Food Sci. Technol.* **2015**, *50*, 2639–2643, doi:10.1111/ijfs.12934.
15. Campana, R.; Baffone, W. Carvacrol efficacy in reducing microbial biofilms on stainless steel and in limiting re-growth of injured cells. *Food Control.* **2018**, *90*, 10–17, doi:10.1016/j.foodcont.2018.02.029.
16. Engel, J.B.; Heckler, C.; Tondo, E.C.; Daroit, D.J.; da Silva, M.P. Antimicrobial activity of free and liposome-encapsulated thymol and carvacrol against Salmonella and Staphylococcus aureus adhered to stainless steel. *Int. J. Food Microbiol*. **2017**, *252*, 18–23, doi:10.1016/j.ijfoodmicro.2017.04.003.
17. Ren, D.; Zuo, R.; Barrios, A.F.G.; Bedzyk, L.A.; Eldridge, G.R.; Pasmore, M.E.; Wood, T.K. Differential gene expression for investigation of Escherichia Coli biofilm inhibition by plant extract ursolic acid. *Appl. Environ. Microbiol*. **2005**, *71*, 4022–4034, doi:10.1128/AEM.71.7.4022-4034.2005.
18. Hu, J.; Garo, E.; Goering, M.G.; Pasmore, M.; Yoo, H.; Esser, T.; Sestrich, J.; Cremin, P.A.; Hough, G.W.; Perrone, P. Bacterial biofilm inhibitors from Diospyros dendo. *J. Nat. Prod*. **2006**, *69*, 118–120, doi:10.1021/np049600s.
19. Bag, A.; Chattopadhyay, R.R. Synergistic antibacterial and antibiofilm efficacy of nisin in combination with p-coumaric acid against food-borne bacteria Bacillus cereus and Salmonella typhimurium. *Lett. Appl. Microbiol*. **2017**, *65*, 366–372, doi:10.1111/lam.12793.
20. Lin, L.; Wang, J.; Yu, J.; Li, Y.; Liu, G. Effects of allicin on the formation of Pseudomonas aeruginosa biofilm and the production of quorum-sensing controlled virulence factors. *Pol. J. Microbiol*. **2013**, *62*, 243–251, doi:10.33073/pjm-2013-032.
21. Pérez-Giraldo, C.; Cruz-Villalón, G.; Sánchez-Silos, R.; Martínez-Rubio, R.; Blanco, M.; Gómez-García, A. In vitro activity of allicin against Staphylococcus epidermidis and influence of subinhibitory concentrations on biofilm formation. *J. Appl. Microbiol*. **2003**, *95*, 709–711, doi:10.1046/j.1365-2672.2003.02030.x.
22. Dürig, A.; Kouskoumvekaki, I.; Vejborg, R.M.; Klemm, P. Chemoinformatics-assisted development of new anti-biofilm compounds. *Appl. Microbiol. Biotechnol*. **2010**, *87*, 309–317, doi:10.1007/s00253-010-2471-0.
23. Brackman, G.; Hillaert, U.; van Calenbergh, S.; Nelis, H.J.; Coenye, T. Use of quorum sensing inhibitors to interfere with biofilm formation and development in Burkholderia multivorans and Burkholderia cenocepacia. *Res. Microbiol*. **2009**, *160*, 144–151, doi:10.1016/j.resmic.2008.12.003.
24. Ding, X.; Yin, B.; Qian, L.; Zeng, Z.; Yang, Z.; Li, H.; Lu, Y.; Zhou, S. Screening for novel quorum-sensing inhibitors to interfere with the formation of Pseudomonas aeruginosa biofilm. J*. Med. Microbiol*. **2011**, *60*, 1827–1834, doi:10.1099/jmm.0.024166-0.
25. Artini, M.; Papa, R.; Barbato, G.; Scoarughi, G.; Cellini, A.; Morazzoni, P.; Bombardelli, E.; Selan, L. Bacterial biofilm formation inhibitory activity revealed for plant derived natural compounds. *Bioorg. Med. Chem.* **2012**, *20*, 920–926, doi:10.1016/j.bmc.2011.11.052.
26. Miladinović, D.L.; Ilić, B.S.; Kocić, B.D.; Marković, M.S.; Miladinović, L.C. In vitro trials of Dittrichia graveolens essential oil combined with antibiotics. *Nat. Prod. Commun.* **2016**, *11*, 865–868.
27. Silva, V.A.; Sousa, J.P.; Guerra, F.Q.S.; Pessôa, H.L.F.; Freitas, A.F.R.; Coutinho, H.D.M.; Alves, L.B.N.; Lima, E.O. Antibacterial activity of the monoterpene linalool: Alone and in association with antibiotics against bacteria of clinical importance. *Int. J. Pharmacogn. Phytochem. Res.* **2015**, *7*, 1022–1026.
28. Miladi, H.; Zmantar, T.; Kouidhi, B.; Al Qurashi, Y.M.A.; Bakhrouf, A.; Chaabouni, Y.; Mahdouani, K.; Chaieb, K.. Synergistic effect of eugenol, carvacrol, thymol, p-cymene and gamma-terpinene on inhibition of drug resistance and biofilm formation of oral bacteria. *Microb. Pathog.* **2017**, *112*, 156–163, doi:10.1016/j.micpath.2017.09. 057.
29. Dwivedi, G.Raj.; Maurya, A.; Yadav, D.K.; Singh, V.; Khan, F.; Gupta, M.K.; Singh, M.; Darokar, M.P.; Srivastava, S.K. Synergy of clavine alkaloid ‘chanoclavine’ with tetracycline against multi-drug-resistant E. coli. *J. Biomol. Struct. Dyn.* **2019**, *37*, 1307–1325, doi:10.1080/07391102.2018.1458654.
30. Lima, V.N.; Oliveira-Tintino, C.D.M.; Santos, E.S.; Morais, L.P.; Tintino, S.R.; Freitas, T.S.; Geraldo, Y.S.; Pereira, R.L.S.; Cruz, R.P.; Menezes, I.R.A.; et al. Antimicrobial and enhancement of the antibiotic activity by phenoliccompounds: Gallic acid, caffeic acid and pyrogallol. *Microb. Pathog.* **2016**, *99*, 56–61, doi:10.1016/j.micpath.2016.08.004.
31. Lin, R.D.; Chin, Y.P.; Hou, W.C.; Lee, M.H. The effects of antibiotics combined with natural polyphenols against clinical methicillin-resistant Staphylococcus aureus (MRSA). *Planta Med*. **2008**, *74*, 840–846. https://doi.org/10.1055/s-2008-1074559.
32. Holler, J.G.; Christensen, S.B.; Slotved, H.-C.; Rasmussen, H.B.; Guzman, A.; Olsen, C.-E.; Petersen, B.; Mølgaard, P. Novel inhibitory activity of the Staphylococcus aureus NorA efflux pump by a kaempferol rhamnoside isolated from Persea lingue Nees. *Antimicrob. Chemother*. **2012**, *67*, 1138–1144, doi:10.1093/jac/dks005.
33. de Araújo, R.S.A.; Barbosa-Filho, J.M.; Scotti, M.T.; Scotti, L.; da Cruz, R.M.D.; Falcão-Silva, V.d.S.; de Siqueira-Júnior, J.P.; Mendonça-Junior, F.J.B. Modulation of drug resistance in Staphylococcus aureus with coumarin derivatives. *Scientifica* **2016**, *2016*, 1–6. , doi:10.1155/2016/6894758.
34. Abulrob, A.N.; Suller, M.T.; Gumbleton, M.; Simons, C.; Russell, A.D. Identification and biological evaluation of grapefruit oil components as potential novel efflux pump modulators in methicillin-resistant Staphylococcus aureus bacterial strains. *Phytochemistry* **2004**, *65*, 3021–3027, doi:10.1016/j.phytochem.2004.08.044.
35. Bazzaz, B.S.F.; Memariani, Z.; Khashiarmanesh, Z.; Iranshahi, M.; Naderinasab, M. Effect of galbanic acid, a sesquiterpene coumarin from Ferula szowitsiana, as an inhibitor of efflux mechanism in resistant clinical isolates of Staphylococcus aureus. *Braz. J. Microbiol*. **2010**, *41*, 574–580, doi:10.1590/S1517-83822010000300006.

Siriwong, S.; Teethaisong, Y.; Thumanu, K.; Dunkhunthod, B.; Eumkeb, G. The synergy and mode of action of quercetin plus amoxicillin against amoxicillin‐resistant Staphylococcus epidermidis. *BMC Pharmacol. Toxicol.* **2016**, *17*, 39, doi:10.1186/s40360-016-0083-8.
